# Supplementary material for: Anthropometric Measurements and Admission Parameters as Predictors of Acute Respiratory Distress Syndrome in Hospitalized COVID-19 Patients
Source: Biomedicines. 2023 Apr 18;11(4):1199. doi: 10.3390/biomedicines11041199 (PMC10135448; doi:10.3390/biomedicines11041199)
Supplement: Supplementary file 1 [file biomedicines-11-01199-s001.zip › biomedicines-2279979-supplementary.pdf]

Supplementary Table S1. Flowchart of patient enrolment

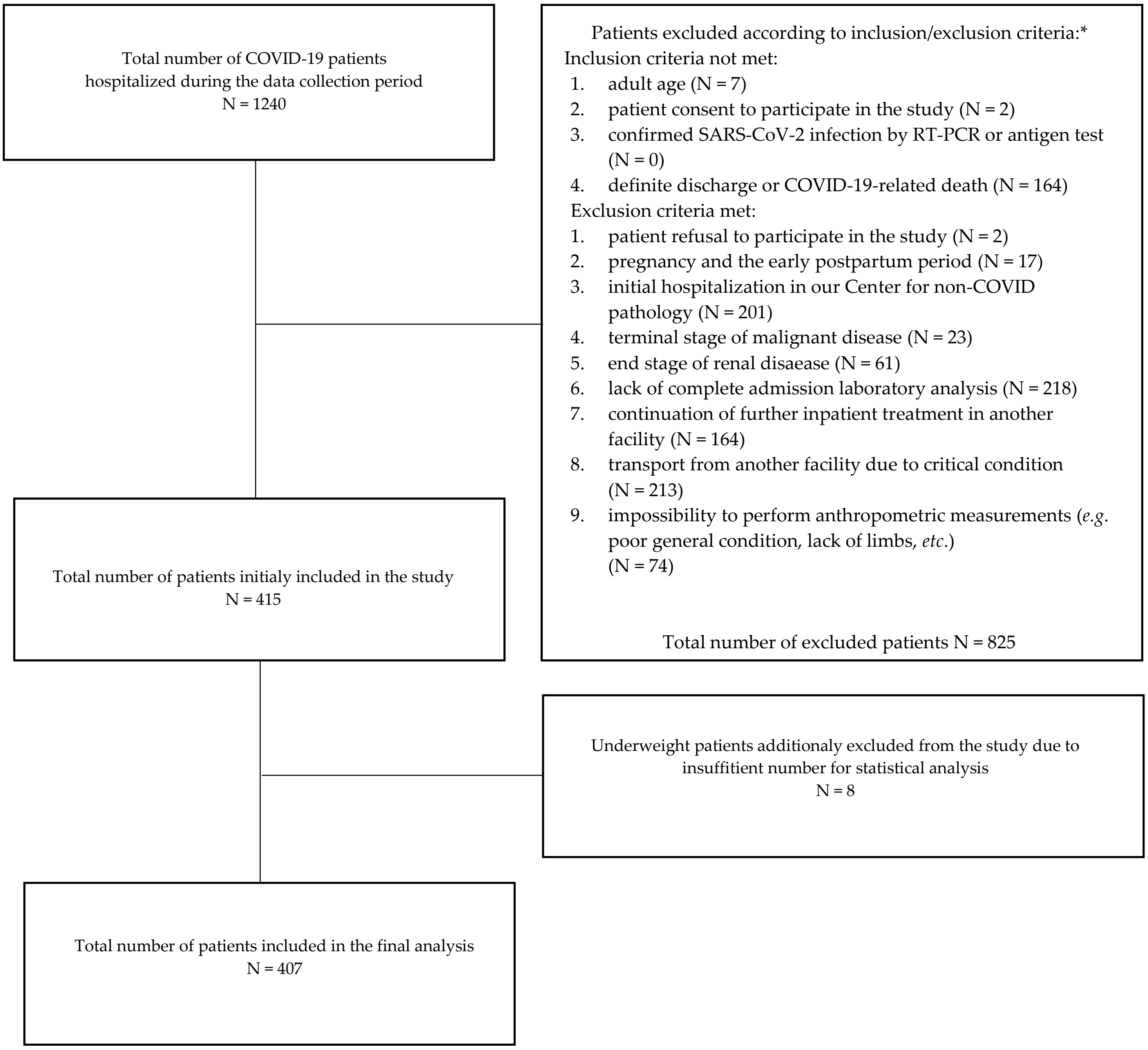

* some patients had more than one exclusion criteria present.

Supplementary Table S2. Threshold values for continuous variables, according to the ROC analysis or laboratory reference lines.

Parameter	Threshold Value	AUC (CI)	„*p*" value
SaO2 [%]	87.5	0.763 (0.713-0.812)	< 0.001*
PaO2 [kPa]	6.85	0.718 (0.665-0.771)	< 0.001*
Lymphocytes [10^9/L]	1.2	*Laboratory reference line*	
LDH [U/L]	793.5	0.667 (0.612-0.723)	< 0.001*
CK [U/L]	171	*Laboratory reference line*	
CRP [mg/L]	107.5	0.578 (0.520-0.636)	0.009*
Age [years]	68.5	0.582 (0.524-0.640)	0.006*
IL-6 [pg/mL]	59.75	0.639 (0.584-0.694)	< 0.001*

Abbreviations: AUC - area under the curve; CI - confidence interval; CK - creatine kinase; CRP - c reactive protein; IL-6 - interleukin 6; LDH - lactate dehydrogenase; PaO2 - Partial pressure of oxygen; ROC - receiver operating characteristics curve; SaO2 - oxygen saturation of blood;
* - statistical significance level at < 0.05.
